# Integrating oral and social factors in individual caries risk assessments in preschool children—a registry-based study

**DOI:** 10.1007/s40368-024-00928-0

**Published:** 2024-08-05

**Authors:** A. I. Hultquist, A. Levinsson, A. Robertson, N. Sabel

**Affiliations:** 1 Department of Pediatric Dentistry, Institute of Odontology, Sahlgrenska Academy, University of Gothenburg, Box 450, 405 30 Gothenburg, Sweden; 2 Public Dental Service, Region Östergötland, Linköping, Sweden; 3 Centre de Recherche du Centre Hospitalier de L, Université de Montréal, Montréal, Québec Canada; 4 Department of Epidemiology and Biostatistics, School of Population and Global Health, McGill University, Montréal, Québec Canada; 5 School of Public Health and Community Medicine, Sahlgrenska Academy, Gothenburg University, Gothenburg, Sweden

**Keywords:** Dental caries susceptibility, Oral health, Dentistry for children

## Abstract

**Purpose:**

To investigate the predictive ability of individual Caries Risk Assessments (CRA) regarding oral factors supplemented with social factors in relation to caries outcome in preschool children. Furthermore, to assess various models of CRA with oral and social factors included, aiming to identify the most suitable models for different age groups.

**Methods:**

The design is a retrospective registry-based cohort study. Children visiting the dentists at ages 3 and 6 years were included. Data on oral and social factors were obtained from dental records, the Swedish Quality register for caries and periodontitis (SKaPa), and Statistics Sweden (SCB).

Various models of CRA were designed, combining oral and social factors. Models were analyzed with univariable associations using simple logistic regression, and the results were presented as odds ratios (ORs). In addition, models were analyzed with area under the receiver operating characteristic (ROC) curve (AUC). Pairwise comparisons were conducted by DeLong’s test, with *p* < 0.05 considered significant.

**Result:**

Oral factors were the most significant for caries outcome (OR 9.6), followed by social factors: foreign background (OR 4.6), low income (OR 2.83), low education of the mother (OR 2.77), single-parent family (OR 2.11), and having ≥ 3 siblings (OR 1.71), (*p* < 0.01).

The predictive ability of CRA improved when models combining oral and social factors were used, compaired to CRA based solely on oral factors (*p* < 0.05). An increase of up to 15% was seen when CRA was conducted closer to the outcome.

**Conclusion:**

Models for Caries Risk Assessment including oral and social factors increase the predictive ability. Caries Risk Assessment has limited durability.

## Introduction

Assessing caries risk and addressing caries in preschool children is a complex issue. Caries is a significant public dental health concern, both in developing and industrialized nations (WHO 2023). Caries remains a global challenge for both children and adults (Dye 2017). The National Board of Health and Welfare report shows a 5% caries incidence among 3-year-olds and a 25% incidence among 6-year-olds in Sweden, with variations observed between and within regions (National Board of Health and Welfare. Caries Prevalence in children 2021). When a child experiences caries, it not only affects their quality of life but also has repercussions for the family in terms of guilt and anxiety (Sabel et al. 2023; Tinanoff et al. 2019; Zaror et al. 2022). Children who suffer from caries in their primary teeth also have a high risk of their permanent teeth being affected later in life (Saethre-Sundli et al. 2020; Tinanoff et al. 2019). This underscores the necessity for a reliable and validated Caries Risk Assessment (CRA) method to prevent caries development in young children.

Caries is a multifactorial disease influenced by biologic, social, and environmental factors (Featherstone et al. 2021). Whitehead and Dahlgren described the *Determinants of health* as the influence of various environmental levels on health, which also impacts the development of caries disease (Whitehead & Dahlgren 1991). Risk indicators for caries in children encompass factors such as culture, foreign background, and family socioeconomics (related to education, finances, and health literacy), which shape individuals’ behaviors and lifestyles (Anderson et al. 2021; Featherstone et al. 2021; Julihn et al. 2018; Yousaf et al. 2022). Previously published studies reported that risk assessment sensitivity for different age groups and geographic regions should emphasize the need for validation within the specific population where it is to be applied (Mejare et al. 2014).

Further studies have described the association between social factors and caries in preschool children at a group level (Anderson et al. 2021; Julihn et al. 2020; Yousaf et al. 2022). Julihn identified a relationship between birth order and caries risk at 7 years of age, with a higher birth order associated with a higher risk of caries (Julihn et al. 2020). However, the findings of these studies cannot be generalized to an individual level as they do not address individual CRA, but rather the presence of caries (Anderson et al. 2021; Julihn et al. 2020; Yousaf et al. 2022).

In CRA, it is crucial to consider known oral caries risk and protective factors, general health, the presence of caries, and social factors (Antunes et al. 2018; Featherstone et al. 2021). In a theoretical patient study, Featherstone combined oral and social factors to optimize the prognosis of caries development in CRA (Featherstone et al. 2021).

CRA on an individual level is essential for anticipating caries development. It facilitates the planning of interventions focused on preventing lesion progression, primary prevention, frequency of interventions, and scheduling recalls (Evans et al. 2018).

During recalls, CRA must be evaluated. If the risk was declared and no caries has developed, the CRA is deemed “incorrect” be appears inconsistent (Twetman 2016). This inconsistency complicates the refinement of CRA, and the prophylaxis treatment initiated by the CRA will affect the outcome. It is unethical not to plan preventive treatment with a rationale to evaluate the CRA. Nevertheless, ongoing improvement and refinement of the CRA may result in identifying more children at risk of caries (Twetman 2016).

To the authors’ knowledge, there is limited literature available regarding individual CRA methods for preschool children that consider a combination of oral factors (such as plaque, caries, diet habits, use of fluorides) and social factors (Agouropoulos et al. 2022; Antunes et al. 2018; Martignon et al. 2021). Therefore, the aim was to investigate the predictive ability of individual CRA regarding oral factors supplemented with social factors, in relation to caries outcome in preschool children. Furthermore, the study aimed to analyze various models that incorporate both oral and social factors to identify the most suitable models for different age groups.

## Material and method

The study design comprises a retrospective registry-based cohort study.

### Material

The cohort includes preschool children who underwent dental examinations at both 3 and 6 years of age at the Public Dental Service, Region of Kalmar Council, located in the south of Sweden.

### Data collection

Data was obtained from dental records, the Swedish Quality register for caries and periodontitis (SKaPa), and Statistics Sweden (SCB). Individual information was tracked using security numbers.

### Caries risk assessment

Initially, data pertaining to CRA, R2, were collected from the individual dental records in the cohort. R2 is an algorithmic tool for assessing the risk of caries in preschool children and adults. This tool incorporates information from dental records, encompassing both manifest and initial caries (excluding those on occlusal surfaces). Examiners voluntarily supplement details such as diet habits, oral hygiene, medical risks, and the use of fluorides. The caries status and modifying variables yields various oral health profiles, classifying individuals into three levels of caries risk: low, moderate, or high (Staberg et al. 2016). Refer to Table 1 for an overview of R2. In this study, R2 is further specified as *oral factors*.

**Table 1:** R2 guidelines for caries risk assessment used by Region of Kalmar

Low caries risk group	Moderate caries risk group	High caries risk group
No caries activity (no new surfaces with caries, inactive initial caries lesions)	1–2 new surfaces with caries in active areas, progression of initial caries	Progression of several initial caries surfaces
Low caries progression in recent years	Moderate caries progression in recent years	≥ Three new surfaces with caries, high caries progression in recent years
Good diet and drinking habits	Cariogenic diet with moderate intake	Cariogenic diet with high intake
Good oral hygiene, PI 0–20%	Oral hygiene, PI 20–50%	Oral hygiene, PI > 50%
Good salivary function and no medication affecting saliva production	Experience of saliva reduction and/or medication affecting saliva production	Dry mouth and medication affecting saliva production
Fluoride supplement for patients’ need	Less fluoride supplement than patients’ need	Less fluoride supplement than patients’ need

### Caries status

Subsequently, the individuals’ social security numbers, along with the R2 obtained from dental records, were transmitted to SKaPa for additional data collection. The data from SKaPa at the individual level pertained to registrations of dental examinations and the status of caries on an annual basis, spanning ages 3 to 6 years. SKaPa employs a national code system to document the status and treatments (*The Swedish Quality Registry for Caries and Periodontal Diseases*, The Swedish Quality Registry for Caries and Periodontal Diseases). The data is sourced from digital dental record systems, providing the possibility to monitor the status and treatments conducted at the individual level over time.

The status of caries in primary teeth is recorded as deft, wherein ‘d’ denotes decayed manifest caries, ‘e’ extraction, ‘f’ represents filled, and ‘t’ indicates the total number of teeth.

### Statistics Sweden

The data files were securely transmitted to SCB. The data collected from SCB at the individual level included factors associated with various aspects of the family, parents, and the socioeconomic situation, Table 2. These aspects are further categorized as social factors.

**Table 2:** Table over analyzed variables, description, and outcome

Variables	Register	Description	Outcome
Caries^a^	SKaPa^b^	Deft (decayed, extracted, filled teeth)	Deft = 0 Deft > 0
Risk assessment R2^a^	Dental record	Low Moderate and high risk	Non-risk Risk
Ethnic background, child^c^	Statistics Sweden	Swedish born with two Swedish born parents Foreign born/ Swedish born with one or two foreign born parents, non-Nordic country	Swedish background Foreign background
Siblings^c^	Statistics Sweden	Presence of siblings	Siblings = 0 Siblings > 0
Siblings^c^	Statistics Sweden	Number of siblings	Siblings < 3 Siblings ≥ 3
Siblings^c^	Statistics Sweden	Birth order	Younger siblings Younger and older siblings = middle child Older siblings
Years in free preschool^d^	Statistics Sweden	Preschool years	= 3 years < 3 years
Changing addresses^d^	Statistics Sweden	Numbers of moving	0–3 > 3
Residential area^e^	Statistics Sweden	Home situation	Outside urban area Urban area Central municipal
Family situation^e^	Statistics Sweden	Family constellation	Two parents Single-parent family Other family constellation
Age of mother/father at child’s birth^c^	Statistics Sweden	23 to 39 years old < 23 and > 39 years old	Middle-aged parents Younger and older parents
Mother’s/Father’s years of education^e^	Statistics Sweden	≤ 9 years of education 10–12 years of education > 12 years education	Uncompleted and completed elementary school Completed upper secondary school University
Equalized disposable income^e,f^	Statistics Sweden	60% of median income for: 2015 2016 2017	Income under or above: 143 200 SEK^g^ 146 000 SEK^g^ 149 600 SEK^g^

^a^Data collected annually 2015–2018

^b^Swedish Quality register for caries and periodontitis

^c^Data collected 2015

^d^Data collected 2018

^e^Data collected 2015–2017

^f^Equalized disposable income: Includes the total income the family disposes after taxes and regulated for how many members who are living together, including children and ages of family members. Equalized disposable income is reported as income under or above the limit of 60% median income. Sixty percent of median income is a limit for low economic standard and at risk of poverty

^g^Swedish crowns

Subsequently, the data files were returned in a de-identified format to researchers for analysis. The variables concerning oral and social factors, along with descriptions and outcomes of the analyzed data, are outlined in (Table 2).

### Statistics

The caries status assessed at 3 years of age is denoted as deft_3,_ and at 6 years of age it is labeled deft_6._

Prior to analysis, moderate and high caries risks from R2 were combined into *risk*–forming two subgroups: *no risk* and *risk.* R2 was performed annually between 3 and 6 years of age. At 3 years of age, R2 is defined as R2_3_; when conducted at 4 or/and 5 years, it is named R2_4&5_*.* R2_3_ and R2_4&5_ were followed up at 6 years of age, categorizing outcomes into having caries (deft_6_ > 0) or not having caries (deft_6_ = 0). Correct *risk* assessment was determined when caries was present at 6 years of age, while a *no risk* assessment was deemed correct when caries was absent at 6 years of age.

Data analysis was conducted using Stata Release 17.0 SE-Standard Edition, Stata Corp LLC. Stata Corp. The dependent variable was defined as deft > 0 at 6 years of age. Univariable associations between the outcome and independent variables were estimated through simple logistic regression, reported as odds ratios (ORs), 95% confidence intervals (CIs), and p-values. *p*- values < 0.05 were considered statistically significant. In subsequent steps, factors identified as statistically significant in univariable models were combined into multivariable models, and the best-fitting model for predicting caries at 6 years of age was selected. Model fit was assessed using the receiver operating characteristic (ROC) Area under the ROC Curve (AUC).

Various risk models were analyzed using the (ROC) area under the curve (AUC). Model A, exclusively incorporating *R2, oral factors*, was appointed as model A_3_ for oral factors at 3 years age, and model A_4&5_ for oral factors at 4 and 5 years of age. Additionally, nine models were created for both age groups, models B_3_ to J_3_ and B_4&5_ to J_4&5,_ featuring oral factors supplemented with social factors (Tables 3, 4).

**Table 3:** Overview of models and the included combinations of oral and social factors at 3 years of age (*n* = 667)

Variables	Models									
	A_3_	B_3_	C_3_	D_3_	E_3_	F_3_	G_3_	H_3_	I_3_	J_3_
Oral factors										
Risk at age 3	x	x	x	x	x	x	x	x	x	x
Social factors										
Foreign background (non-Nordic country)		x	x	x	x	x			x	x
Siblings										
≥ 3 siblings at 3 years		x	x					x	x	x
Family situation										
Single-parent family at 3 years age			x	x			x	x	x	
Education parents										
Father uncompleted/completed elementary school at 3 years					x	x	x	x	x	x
Mother uncompleted/completed elementary school at 3 years						x	x	x	x	
AUC	0.63	0.69*	0.70*	0.68*	0.66	0.68*	0.68*	0.70*	0.70*	0.69*

^*^Pairwise comparison to model A_3_ (DeLong test *p* < 0.05), all models having larger AUC compared to A_3_

The areas under the receiver operating characteristic (ROC) curve (AUC) are given for each model in relation to outcome deft > 0 at 6 years of age

**Table 4:** Overview of models and the included combinations of oral and social factors at 4&5 years of age (*n* = 435)

Variables	Models									
	A_4&5_	B_4&5_	C_4&5_	D_4&5_	E_4&5_	F_4&5_	G_4&5_	H_4&5_	I_4&5_	J_4&5_
Oral factor										
Risk at ages 4 & 5	x	x	x	x	x	x	x	x	x	x
Social factors										
Foreign background (non-Nordic country)		x	x	x	x	x			x	x
Siblings										
≥ 3 siblings at age 3		x	x					x	x	x
Family situation										
Single-parent family at 4&5 years of age			x	x			x	x	x	
Education parents										
Father uncompleted /completed elementary school at child’s age 4&5					x	x	x	x	x	x
Mother uncompleted /completed elementary school at child’s age 4&5						x	x	x	x	
AUC	0.78	0.80*	0.82*	0.81*	0.81*	0.81*	0.80*	0.81*	0.82*	0.82*

^*^Pairwise comparison to model A_4&5_ showed larger AUC (DeLong test *p* < 0.05)

The areas under the receiver operating characteristic (ROC) curve (AUC) are given for each model in relation to outcome deft > 0 at 6 years of age

Pairwise comparison of ROC curves between models and within specific age groups were conducted (Tables 3, 4). In addition, pairwise comparisons for models in groups with R2_3_ and R2_4&5_ were conducted between data from ages 3 years and 4 and 5 years using DeLong’s test for statistical assessment.

### Ethical approval

The Swedish Ethical Review Authority approved the study under Dnr 2021–00887, supplemented by 2022–01739-02 and 2022–03206-02.

## Results

### Included children

**Fig. 1:**
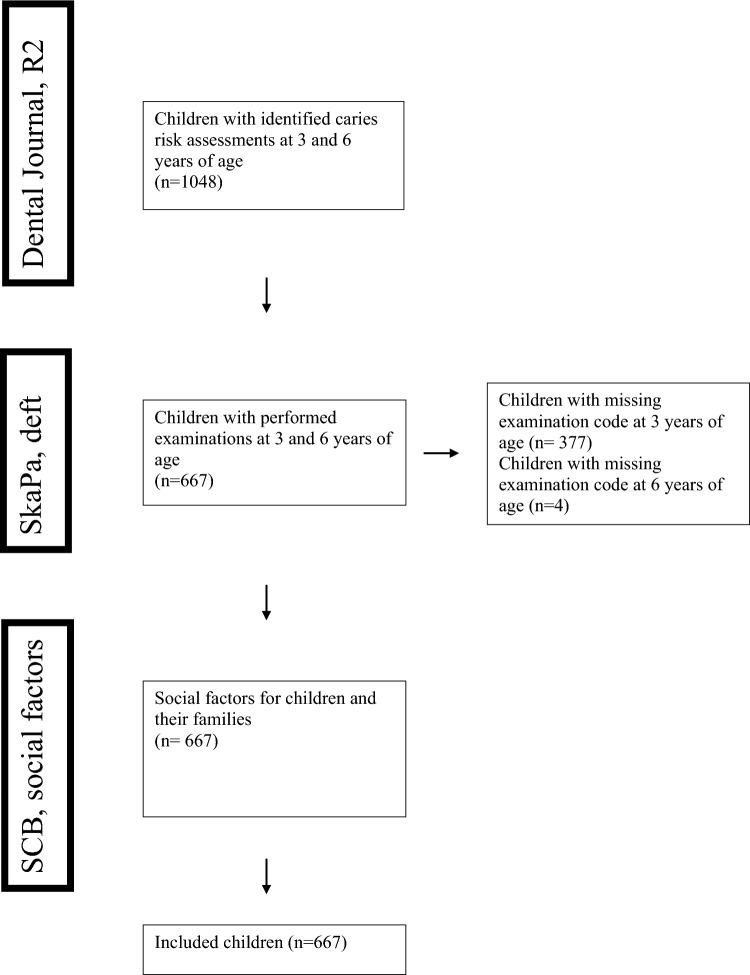
Flow chart detailing the inclusion of children and drop-outs in the study. Reasons for the drop-out of 381 children were attributed to the absence of examination codes

When analyzing dental records from the Public Dental Service, 1048 children (510 girls, 538 boys) meeting the criteria were identified, all of whom had CRAs at 3 and 6 years of age. Data from SKaPa showed that 381 children were lacked an examination code and were consequently excluded from further analysis (Fig. 1). The remaining 667 children, comprising 330 (49%) girls and 337 (51%) boys, were included in the subsequent study. Among these, 435 children also underwent R2_4&5._

### Caries status

At 3 years of age, 41 children (6%), including 22 girls and 19 boys, exhibited signs of caries with deft_3_ > 0. By 6 years of age, deft_6_ > 0 was observed in 169 children (25%), comprising 81 girls and 88 boys.

The mean (± SD) for deft at 3 years (deft_3_) was 0.23 (± 1.16), and at 6 years, deft_6_ was 0.96 (± 2.17).

### Caries risk assessment–oral factors

When analyzing R2, R2_3_ showed 37 children (6%) at risk for caries, while R2_4&5_ resulted in 79 children (13%) were at risk (Table 5).

**Table 5:** Results from caries risk assessment, proportion, and simple regression analyses of the associations between oral and social factors (from Table 2), and the prevalence of caries (deft > 0) at 6 years of age

Variables	Total *n* (%)	Caries at 6 years age *n* (%)	OR	*p*	Simple log reg 95% CI
Boys	337 (51)	88 (13)	1.09	0.642	0.77–1.54
Girls	330 (49	81 (12)			
Oral factors (R2)					
Caries risk at 3	37 (6)	26 (70)	9.60	< 0.0001	4.48–20.46
Caries risk at ages 4&5	79 (13)	67 (85)	36.71	< 0.0001	18.47–72.95
Social factors					
Foreign background (non-Nordic country)	111 (17)	59 (53)	4.60	< 0.0001	3.00–7.05
Siblings					
Siblings at age 3	639 (96)	161 (25)	1.20	0.002	1.08–1.43
≥ 3 siblings at age 3	132 (20)	45 (34)	1.71	0.010	1.14–2.59
Older siblings at age 3	397 (59)	101 (27)	1.1	0.536	0.78–1.60
Younger siblings at age 3	375 (59)	101 (27)	1.18	0.371	0.82–1.67
Middle child at age 3	133 (21)	43 (32)	1.55	0.039	1.02–2.34
Years in preschool > 2 at age 5	638 (96)	153 (24)	0.22	0.001	0.09–0.52
Residential area					
Outside urban area at age 3	218 (33)	63 (29)	1.3	0.141	0.91–1.89
Urban area at age 3	168 (25)	33 (20)	0.65	0.051	0.42–1.00
Central municipal at age 3	281 (42)	73 (26)	1.06	0.745	0.75–1.51
Outside urban area at ages 4&5	142 (33)	40 (28)	1.16	0.517	0.74–1.82
Urban area at age 4&5	122 (28)	28 (20)	0.79	0.336	0.48–1.28
Central municipal at age 4&5	171 (39)	46 (27)	1.06	0.791	0.69–1.64
> 3 Changing addresses btw 3 and 6 years	7 (1)	2 (29)	1.2	0.843	0.23–6.14
Family situation					
Two-parent family at age 3	578 (87)	129 (22)	0.35	< 0.0001	0.22–0.56
Single-parent family at age 3	55 (8)	22 (40)	2.11	0.010	1.19–3.73
Other family constellation at age 3	34 (5)	18 (53)	3.60	< 0.0001	1.79–7.22
2-parent family at ages 4&5	360 (83)	85 (24)	0.49	0.008	0.29–0.83
Single-parent family at ages 4&5	50 (11)	17 (34)	1.52	0.185	0.82–2.87
Other family constellation at ages 4&5	25 (6)	12 (48)	2.79	0.014	1.23–6.30
Age parents					
Younger or older father	100 (15)	35 (35)	1.76	0.015	1.12–2.78
Middle-aged father	564 (85)	131 (23)	0.57	0.015	0.36–0.89
Younger or older mother	65 (10)	21 (32)	1.46	0.176	0.84–2.54
Middle-aged mother	602 (90)	148 (25)	0.68	0.176	0.39–1.19
Education parents					
Father uncompleted /completed elementary school 2015 at child’s age of 3	79 (12)	33 (42)	2.43	< 0.0001	1.49–3.96
Father completed upper secondary school at child’s age of 3	392 (59)	99 (25)	1.03	0.877	0.72–1.47
Father university level at child’s age of 3	192 (29)	34 (18)	0.55	0.006	0.36–0.84
Father uncompleted /completed elementary school at child’s age of 4&5	49 (11)	21 (43)	2.41	0.005	1.30–4.44
Father completed upper secondary school at child’s age of 4&5	253 (58)	66 (26)	1.02	0.928	0.66–1.58
Father university level at child’s age of 4&5	130 (30)	25 (6)	0.59	0.038	0.36–0.97
Mother uncompleted /completed elementary school at child’s age of 3	59 (9)	27 (46)	2.77	< 0.0001	1.60–4.78
Mother completed upper secondary school at child’s age of 3	321 (48)	89 (28)	1.28	0.172	0.90–1.81
Mother university level at child’s age of 3	287 (43)	53 (18)	0.52	< 0.0001	0.36–0.75
Mother uncompleted /completed elementary school at child’s age of 4&5	30 (7)	14 (47)	2.66	0.011	1.25–5.64
Mother completed upper secondary school at child’s age of 4&5	191 (44)	59 (31)	1.53	0.053	0.99–2.35
Mother university level at child’s age of 4&5	193 (44)	38 (20)	0.53	0.006	0.34–0.83
Income					
Income under limit 60% at child’s age of 3	133 (20)	57 (43)	2.83	< 0.0001	1.89–4.22
Income under limit 60% at child’s age of 4&5	86 (20)	34 (40)	2.20	0.002	1.33–3.62

Odds ratio (OR) > 1 shows a positive association to caries prevalence at 6 years of age. The statistical method for calculating OR and *p* value was simple logistic regression

The presence of *risk* at R2_3_ or R2_4&5_ was significantly associated with caries at 6 years of age (simple regression, *p* < 0.0001). The OR for developing caries at 6 years of age was calculated to be 9.6 for those identified at risk at 3 years and 36.7 for those at risk at 4&5 years of age.

### Social factors

Data from SCB regarding social factors (Table 2) showed varying associations (OR) with caries. Key social factors found to be significantly associated with caries included having a foreign background, low disposable income, a low education level of mother, belonging to a single-parent family, and having ≥ 3 siblings (Table 5).

After analyzing data from dental records, SKaPa, and SCB, it was observed that oral factors, social factors, and a combination of both were associated with caries, with OR > 1 (Table 5). Considering a combination of oral and social factors, various caries risk models were developed based on oral factors and different social factors (Table 5). Models including a foreign background, single parent family, parents’ education level, and having ≥ three siblings were created and analyzed. The predictive ability increased when models included a foreign background, having ≥ three siblings, parents’ education level and single-parent family. In contrast, including economic factors in the created models did not result in any further difference (Tables 3, 4).

The best-fitting models, including both oral and social factors, are presented in Tables 3, 4, represented by the area under the Receiver Operating Characteristic (ROC) Curve (AUC)*.*

### Combined models, 3 years of age

**Fig. 2:**
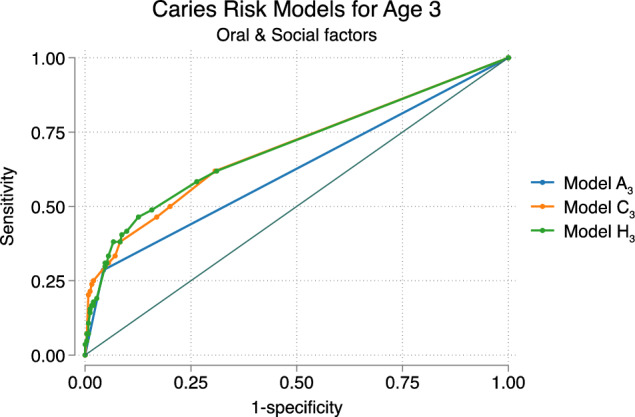
Receiver operating characteristic (ROC) curve depicting the predictive ability of three caries risk models at 3 years of age. Model A_3_ (blue line) includes oral factors with an AUC of 0.63. Model C_3_ (orange line), including oral factors, foreign background, ≥ three siblings, and single-parent family shows an AUC of 0.70. Model H_3_ (green line), including oral factors, ≥ three siblings, single-parent family, and father and mother with low educational levels shows an AUC of 0.70

In models utilizing data from 3 years age (B_3_ to J_3_), the AUC, including both oral and social factors, showed up to a 7% higher prediction compared to oral factors alone in model A_3_. All models showed a higher predictive value with a larger AUC in comparison to model A_3_ (Table 3). Pairwise comparisons of the ROC curves between model A (oral factors) and models B-J (oral and social factors) were conducted, revealing a significant difference except for model E_3_ (DeLong test *p* < 0.05). Pairwise comparisons of the ROC curves among all other models, excluding A, showed no significant difference (DeLong test *p* < 0.05). ROC curves for models A_3_, C_3_, and H_3_ (Fig. 2).

### Combined models, 4&5 years of age

**Fig. 3:**
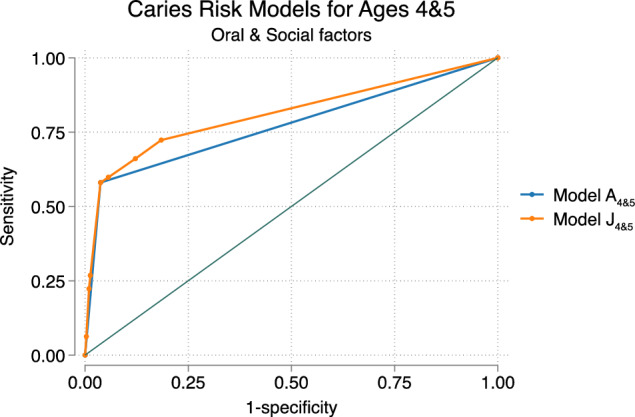
Receiver Operating Characteristic (ROC) curve, depicting the predictive ability of two caries risk models at 4&5 years of age. Model A_4&5_ includes oral factors AUC 0.78, while model J_4&5_ includes oral factors, foreign background, ≥ three siblings, and low educational level of father, AUC 0.82

Analyzing models of data from 4 and 5 years of age (B_4&5_ to J_4&5_), including both oral and social factors with calculations of AUC, resulted in up to a 4% higher prediction compared to model A_4&5_, exclusively including oral factors. All models demonstrated a higher predictive value with a larger AUC compared to model A_4&5_ (Table 4). Pairwise comparisons of the ROC curves between model A (oral factors) and models B-J (oral and social factors) revealed a significant difference between all models (DeLong test *p* < 0.05). Pairwise comparisons of the ROC curves among all other models, excluding A, showed no significant difference (DeLong test *p* < 0.05). For the illustration of ROC curves of models A_4&5_ and J_4&5_ (Fig. 3).

### Comparing R2 from different ages

**Fig. 4:**
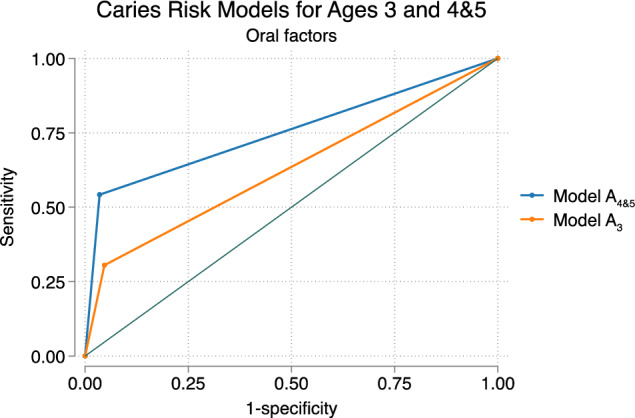
Receiver Operating Characteristic (ROC) curve, depicting the predictive capability of the caries risk model, including oral factors at 3 and 4&5 years of age, model A_3_ AUC 0.63 and model A_4&5_ AUC 0.78

To compare R2_3_ to R2_4&5_, the AUC of models A_3_ and A_4&5_ were calculated. The AUC for A_3_ was 0.63, and for A_4&5_, it was 0.78 (Tables 3, 4). The predictive ability increased 15% when performing R2_4&5_ compared to R2_3_ based on oral factors (Fig. 4).

### Comparing models from different ages

When reviewing the models A to J at the various ages, the prediction increased by 8–15% when utilizing models from 4 and 5 years, comparing AUCs for the different models at different ages. The 15% difference was observed in AUC for A_3_ (0.63), compared to AUCs for models C_4&5_, I_4&5_, and J_4&5_ (0.82) (Tables 3, 4). Pairwise comparisons of the ROC curves of model A (oral factors) to B-J (oral and social factors) were conducted, indicating differences between age groups for all models except models C, D and I (DeLong test *p* < 0.05). This implies that the designed models A, B, E–H, and J increased the prediction for CRA when used at 4&5 years of age, compared to use at 3 years age.

## Discussion

This study indicates an enhanced predictive ability when combining oral factors derived from R2 with social factors encompassing family dynamics and socioeconomic aspects in CRA. Furthermore, the proximity of CRA to the eventual outcome showed to be of concern; notably, CRA performed at 4&5 years of age exhibits a higher predictive ability than compared to CRA performed at 3 years of age for the outcome of caries at 6 years of age. This suggests that CRA has limited durability during the preschool years.

The findings that oral factors exhibit a better predictive value (higher OR) for caries, compared to any single social factor, underscores the significance of individual encounters and oral examinations in achieving optimal caries prediction. The result of the predictive value increased when oral and social factors were combined, suggesting that a preschool child’s vulnerability to caries is influenced by environmental factors, thus emphasizing the need for including social factors in an optimal CRA. This interpretation is in accordance with Featherstone who declared “A combination of all factors that affect caries development should be included in CRA” (Featherstone et al. 2021). Featherstone compared four different caries risk models with fictitious patients with oral and social conditions, lacking follow-up possibilities, whereas this study utilized authentic patients with actual oral and social situations, enabling a more comprehensive comparison of models with included oral and social factors, along with a follow-up of caries status at 6 years (Featherstone et al. 2021).

To emphasize the significance of social factors in predicting dental health at age 3 compared to ages 4&5, the study confirmed that social factors impact the prediction as more pronounced at 3 years of age than at 4&5 years of age. Anderson similarly obtained results consistent with this in a 2021 study, indicating a diminishing impact of social factors as the child’s ageincreases (Anderson et al. 2021). Living in a single-parent family appears to have a more significant impact on predicting dental health outcomes for 3-year-old children than for 4&5-year-olds, a finding supported by Baggio. Baggio confirmed children having poorer dental health often belonged to families with an immigrant background, lower socioeconomic status, parents with lower levels of education, and single-parent families (Baggio et al. 2015).

The addition of the variable “economy” to the best-fitting models did not enhance the predictive value, which contrasts with findings in other studies (Anderson et al. 2021; Baggio et al. 2015; Julihn et al. 2018).

This study illustrates that including social factors in different models increases predictive ability, underscoring the importance of not relying exclusively on oral factors when performing a CRA.

The CRA performed at 4&5 years of age exhibits higher predictive ability than CRA performed at 3 years of age, emphasizing the need for regular and frequent reassessment in early childhood, a result consistent with studies by Mejare and Twetman (Mejare et al. 2014; Twetman 2016).

This study reveals the limited durability of CRA, emphasizing its consistency when performed closer to the follow-up time. As known, CRA provides a snapshot of the patient’s situation at a specific moment susceptible to change over time, thereby necessitating regular reassessments. Numerous studies confirm that within a 1–2-year timeframe, children undergo changes in the risk of developing caries (Jorgensen & Twetman 2020; Tellez et al. 2013; Twetman et al. 2013).

The study’s material is considered representative, encompassing children at both low and high caries risk. The caries prevalence (deft > 0) at 3 years of age in the studied population was 6%, aligning with national data of 4% (National Board of Health and welfare. Caries prevalence in 2015). At 6 years of age, caries prevalence was 25% in the study, while national data showed a prevalence of 27% (National Board of Health and welfare. Caries prevalence in 2018).

One limitation of the study design is that the living areas for the studied population do not include any metropolitan areas. Another limitation may be the number of dental practitioners noted the dental status and registered CRA (R2), but this is reflective of realistic conditions and adheres to clear guidelines from the Region. R2 includes general and oral health, pattern of diet, and oral hygiene routines, and to optimize the study it would be beneficial to register all parameters separately.

Further research is needed, including an analysis of preventive treatments implemented after CRA and the outcomes of such treatments. The potential inclusion of saliva tests and biomarkers could enhance the effectiveness of CRA. In future, Artificial Intelligence (AI) may play a role in guiding individual treatment plans by analyzing risk factors.

## Conclusion

Considering the limitations of the present study it has been shown that the inclusion of both oral and social factors in caries risk assessment increases the predictive ability of the assessment. Utilizing various models incorporating these factors allows for an improved caries risk assessment. Moreover, the durability of caries risk assessment is limited.

## Data Availability

Data can be obtained on request.
